# Years of Life Lost due to exposure: Causal concepts and empirical shortcomings

**DOI:** 10.1186/1742-5573-1-5

**Published:** 2004-12-16

**Authors:** P Morfeld

**Affiliations:** 1 Institute for Occupational and Social Medicine, Cologne University Medical School, 50931 Cologne, Joseph-Stelzmann-Str. 9, Germany; 2 Institute for Occupational Sciences of RAG Aktiengesellschaft, 44369 Dortmund, Hülshof 28, Germany

**Keywords:** years of life lost, effect measurement, counterfactuals, bias

## Abstract

Excess Years of Life Lost due to exposure is an important measure of health impact complementary to rate or risk statistics. I show that the total excess Years of Life Lost due to exposure can be estimated unbiasedly by calculating the corresponding excess Years of Potential Life Lost given conditions that describe study validity (like exchangeability of exposed and unexposed) and assuming that exposure is never preventive. I further demonstrate that the excess Years of Life Lost conditional on age at death cannot be estimated unbiasedly by a calculation of conditional excess Years of Potential Life Lost without adopting speculative causal models that cannot be tested empirically. Furthermore, I point out by example that the excess Years of Life Lost for a specific cause of death, like lung cancer, cannot be identified from epidemiologic data without assuming non-testable assumptions about the causal mechanism as to how exposure produces death. Hence, excess Years of Life Lost estimated from life tables or regression models, as presented by some authors for lung cancer or after stratification for age, are potentially biased. These points were already made by Robins and Greenland 1991 reasoning on an abstract level. In addition, I demonstrate by adequate life table examples designed to critically discuss the Years of Potential Life Lost analysis published by Park et al. 2002 that the potential biases involved may be fairly extreme. Although statistics conveying information about the advancement of disease onset are helpful in exposure impact analysis and especially worthwhile in exposure impact communication, I believe that attention should be drawn to the difficulties involved and that epidemiologists should always be aware of these conceptual limits of the Years of Potential Life Lost method when applying it as a regular tool in cohort analysis.

## Introduction

The most common epidemiological exposure-disease effect measures are based on exposure or disease frequency statistics, like risks or odds. Such frequency statistics focus on the question whether an exposure or disease occurred in a population. This information is used to measure the effect of exposure on disease by comparisons of such statistics. Although these measures have been proven by practice and theory to be useful for this purpose, these frequency statistics are unable to reflect all causal effects of exposure in general (Greenland and Robins 1988 [3], Robins and Greenland 1989 [4]). One reason stems from the fundamental fact that exposure and disease are processes in time. In particular, if time plays a major role in the link between exposure and disease which is certainly true for long-term exposures and chronic diseases, the question when a disease occurs becomes of paramount relevance. It is important to note, although not widely recognised, that the temporal shift of the onset of disease caused by exposure falls beyond the grasp of conventional statistics based on risks or odds, at least in part. And this shortcoming is even true, albeit perhaps counter-intuitive at first glance, when time-dependent incidence rates are analysed by applying sophisticated time-related statistical procedures like Cox modelling with or without adjustment for time-dependent covariates (Rothman and Greenland 1998 [5], Greenland 1999 [6], Morfeld and Piekarski 2001 [7]). An illustrative example of a Cox analysis in which the true probability of causation can not be derived correctly from the hazard ratio estimate due to an incompletely reflected temporal shift of the disease onset is given as an endnote (see endnote 1).

Therefore, alternative measures that focus more directly on the time-shift of events or the time-shift of frequency statistics would be most welcome. One such approach aims at Years of Life Lost (YLL). Interestingly, even in the title of one of the very first articles about Years of Life Lost, Dempsey [8] expressed the opinion that important aspects are missed by frequency statistics that could be well covered by Years of Life Lost methodology. An overview of different explications of the concept of Years of Potential Life Lost (YPLL) was given by Gardner and Sanborn 1990 [9]. Moreover, the authors presented a unifying conceptual framework for all these explications of YPLL. In the past the method of Years of Potential Life Lost was mainly used to describe the impact of different causes of death on the survival of a population. This concept was developed further trying to estimate the health effects of specific exposures like smoking (Quellet et al. 1979 [10], Centers for Disease Control 1989 [11]). For this purpose, excess Years of Potential Life Lost due to exposure (e-YPLL) were calculated in two steps: first, for each age group the number of excess deaths among the exposed was multiplied by the expected remaining years of life at age at death, given no exposure, and second, these products were summed over all age categories.

Recently, this approach was extended by Park et al. 2002 [2] from SMR-based calculations to YPLL-estimates based on Poisson regression models. The authors illustrated the method with the Colorado Plateau Uranium Miners Cohort (Lundin et al. 1971 [12]) estimating Years of Life Lost per years worked as a uranium miner, in particular focusing on premature lung cancer deaths. A further application was published by Bailer et al. 2003 [13] in an analysis of occupational fatal injuries.

However, in a letter to the editor, Morfeld 2003 [14] claimed that the proposed method of estimating excess Years of Life Lost due to exposure is potentially biased in certain settings. The arguments presented were based on the fundamental but abstract article written by Robins and Greenland 1991 [1] who investigated the estimability of expected Years of Life Lost due to a hazardous substance. The critique raised in Morfeld 2003 [14] focused on the unavoidable non-identifiability of the true excess Years of Life Lost due to exposure after conditioning on age. However, the train of thoughts was presented in a rather condensed way, and obviously, the arguments were not easy to digest (compare the response by Park et al. 2003 [15])

Here I try to explain the reasoning in detail. I expand the critique demonstrating additionally that the e-YPLL method is unjustified when applied to specific causes of death like lung cancer – the main topic of Park et al. 2002 [2]. First, a general framework of causal thinking in epidemiology is developed. This provides a background to analyse the validity of estimation procedures. In the second Chapter the most elementary setting is used to introduce the concepts of excess Years of Life Lost (e-YLL), Years of Potential Life Lost (YPLL) and excess Years of Potential Life Lost (e-YPLL). Then I show that e-YPLL is an unbiased estimator of e-YLL when considering all causes of death. In the third Chapter I prove the potential bias of the e-YPLL estimator when conditioning on age. The proof is given in an elementary setting. Moreover, I illustrate this bias by a realistic life table example. The next Chapter deals with the potential bias of e-YPLL when applied to specific causes of death. This potential distortion is proved in a simple epidemiological setting and demonstrated by a realistic life table calculation. All life tables presented are designed to discuss the analysis published by Park et al. 2002 [2]: their potentially biased applications of the Years of Potential Life Lost method specific for lung cancer or conditioned on periods of age. These tables are provided in pdf-format as Additional files 1, 2 and 3. They are based on two spreadsheets that are available as Additional files 4 and 5. The final Chapter deals with generalized scenarios, alternative estimation procedures and a brief discussion of the counterfactual approach to causality, even including a hint at its important link to ontological concepts in quantum mechanics.

To summarize, the main focus of this paper is first, on a new and simple proof of the potential bias of the e-YPLL method, and second, on an illustration of the degree of potential bias involved by realistic life table examples. These examples are constructed to critically discuss the e-YPLL analysis of a cohort of US uranium miners presented by Park et al. 2002 [2]. The demonstrated limitation of the e-YPLL approach was first proven rigorously in Jamie Robins's and Sander Greenland's 1991 [1] breakthrough article on the estimability of expected years of life lost due to a hazardous exposure. My didactic intention is to prove and illustrate the problems involved on a much simpler level of argumentation.

## Analysis

### 1. Causal concepts: setting out a cohort gold standard

The aim of this paper is to scrutinise the validity of an exposure effect statistic. Therefore, a gold standard is needed against which the statistic can be evaluated to identify and to measure potential biases. A comprehensive cohort investigation is the most natural and purposeful epidemiologic approach towards causality when leaving aside all practical obstacles linked to this study design (Rothman and Greenland 1998 [5]). To approximate the theoretical gold standard I suppose such a cohort study as being free of selection biases, information errors, losses to follow up, and inferential problems. However, to define an optimal study scenario, I need to assure additionally that contrasts between response statistics of differently exposed sub-cohorts measure correctly the true effect of exposure. This "no confounding" assumption can be visualised by exchangeable sub-cohorts, the most elementary one consisting of only one subject each.

These differently exposed but exchangeable subjects are assumed to be like ideal twins: if exposure status had been interchanged between both twins the health response of the subject exposed to exposure level A would have been exactly the response of his/her twin exposed to level B and vice versa. This scenario of totally exchangeable twins is at the bottom of the so called "counterfactual" approach to causality (Lewis 1973 [8], Rubin 1974 [18], Maldonado and Greenland 2002 [23]). It idealises the experimental approach often applied in science by carefully preparing test objects as similary as possible. The aim of this preparation is to measure the effect of an independent variable on a dependent variable in a series of experiments as unconfounded as possible. A mathematical outline of this approach was developed by Neyman 1923 [16] and even extended to non-experimental studies by Simon and Rescher 1966 [17], Rubin 1974 [18] and Holland 1986 [19]. Overviews are given by Rosenbaum 1995 [20] and Pearl 2000 [21]. In addition, Robins 1997 [22] applied the counterfactual approach to conceptualise and analyse scenarios with interdependent exposure variables, response variables and other covariates that develop in time. I will come back to a critical discussion of this fundamental approach to causality within the Discussion section. At the moment it is sufficient to understand that the gold standard for epidemiology is set out here as an ideal cohort study where exposure levels are allocated to n-tuples of exchangeable subjects.

Hence, a gold standard scenario with a binary point exposure assumes that each exposed subject has an ideal unexposed twin so that the exposed sub-cohort consists of all the exposed twins and the unexposed sub-cohort comprises all the unexposed twins. Each causal comparison between the sub-cohorts is based on the pairwise comparison of the differently exposed but perfectly exchangeable twins, at least indirectly (Maldonado and Greenland 2002 [23]). Obviously, even randomisation of exposure is unnecessary to gain an unbiased estimate of the exposure effect in such a gold standard study.

Note that the sub-cohort of unexposed twins constitutes an ideal reference population. It is ideal with respect to 1) being an optimal reference group to define the response of the exposed cohort had it not been exposed (comparison on the group level). Over and above this optimality criterion, the reference is ideal with respect to 2) being an optimal reference on the individual level: the response of each exposed subject can be compared to the response of his/her twin. Note that optimality criterion 2) entails optimality criterion 1). Whereas an external reference population as often chosen in concrete epidemiological studies (Rothman and Greenland 1998 [5], Breslow and Day 1987 [24]) can be supposed to fulfil criterion 1), at least approximately, the optimality criterion 2) will not be guaranteed. The reason is that the external reference population cannot provide an unexposed control partner on the individual level. Thus, statistics like SMRs tailored for analysis of such data do not rely on individual level comparison information. It is important to note that these statistics, due to their definition, could not make use of such information even if it were available. Chapter 2 demonstrates that the e-YPLL method can be applied unbiasedly given optimality criterion 2) when applied to all causes of deaths and without any stratification. Chapters 3 and 4 show that e-YPLL stratified on age or specified for certain endpoints, like lung cancer, may be potentially biased even if criterion 2) is assumed. This is so because e-YPLL ignores the information about individual matching. However, this information is crucial as I will demonstrate in Chapters 3 and 4. It follows that a stratified or specified version of e-YPLL is potentially biased if it is used to analyse cohort studies in comparison to an external reference population.

### 2. Years of Life Lost in an ideal study

#### 2.1 Elementary situation with one pair of twins only

**Figure 1:**
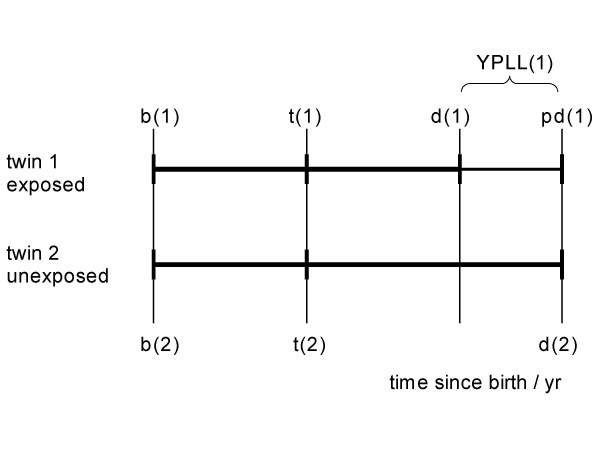
Two exchangeable twins 1 and 2. Birth at b(1) = b(2); allocation of exposure at t(1) = t(2): twin 1 exposed, twin 2 unexposed; death at d(1) ≤ d(2) ; excess years of life lost due to exposure e-YLL = d(2) - d(1); potential death time under no exposure pd(1) = d(2); pd(2) = d(2). Years of potential life lost YPLL(i) = pd(i) - d(i), i = 1,2; excess years of potential life lost e-YPLL = 1(pd(1) - d(1))-0(pd(2)-d(2)) = e-YLL.

In this section I define excess Years of Life Lost (e-YLL) and excess Years of Potential Life Lost (e-YPLL). Then I analyse their relationship in the most simple setting of an ideal study: a pair-matched cohort study with binary point exposure and a death process as outcome. The cohort is assumed to consist of only two subjects, both being differently exposed but totally exchangeable twins. Figure 1 illustrates the scenario.

Both twins (1 and 2) were born on the same day, b(1) = b(2). For simplicity I rescale the time axis and set b(1) = b(2) = 0. Thus, time is identical with age throughout this paper. The point exposure was allocated at an age of t(1) = t(2) > b(1) = b(2) = 0: twin 1 was exposed, twin 2 was never exposed. The twins died at ages d(1) > t(1) and d(2) > t(2), respectively, and deaths are understood as deterministic responses on the individual level. I assume the exposed twin dies earlier or at the same date as the unexposed control twin, d(1) ≤ d(2). Since both subjects are perfectly exchangeable exposure was neutral or detrimental but not preventive. Exposure caused an advancement of death by d(2) - d(1) years. Thus, the true excess Years of Life Lost due to exposure are e-YLL = d(2) - d(1).

Next, I introduce potential death times pd(i), i = 1, 2. If both subjects had been unexposed, I would have expected them to die at pd(i) when relying upon the sub-set of the reference population surviving at least to d(i). Since the ideal reference population consists of the unexposed subject only, it immediately follows pd(1) = d(2) and pd(2) = d(2). For this reason I set pd(1) and d(2) equal in Figure 1. In general, methods like those proposed by BEIR IV 1988 [25] may be applied to the life table of the reference population to estimate pd(i).

I now calculate life expectancy at age at death, YPLL(d(i)), based on the reference population. These Years of Potential Life Lost are defined as YPLL(d(i)) = pd(i) - d(i). Hence, YPLL(d(1)) equals pd(1) - d(1) = d(2) - d(1) = e-YLL in this elementary setting. Obviously I have YPLL(d(2)) = 0. Note that the identity of YPLL(d(1)) and e-YLL relies on the elementary scenario and on the exchangeability condition (together with other usual conditions necessary for study validity).

Next, the effect estimate based on YPLL is introduced. The effect estimate of exposure on health is defined as the excess Years of Potential Life Lost, e-YPLL, which is the sum of excess deaths among the exposed (i.e., the observed exposed deaths minus the expected exposed deaths) times Years of Potential Life Lost at age at death, where the summation runs over all death times t (Park et al. 2002 [2]):

    e-YPLL = Σ (observed(t) - expected(t)) YPLL(t).

Since the number of events is discrete this can always be understood as

    e-YPLL = Σ observed(d(i)) YPLL(d(i)) - Σ expected(d(j)) YPLL(d(j)),

where the first sum is running over all death times d(i) among the exposed twins and the second sum is running over all death times d(j) among the unexposed twins.

The number of expected deaths among the exposed at age t, expected(t), is calculated as the product of the risk among the unexposed at t and the number of exposed at t. This definition is analogous to the calculation of exposed cases in an SMR analysis (Breslow and Day 1987 [24]):

    baseline risk at t = # unexposed events at t / # unexposed twins at t

    expected(t) = (baseline risk at t) (# exposed twins at t).

If there are no unexposed twins at age t, I define expected(t) to be zero.

Two risk sets are given in the simple scenario discussed here: one exposed death occurs at d(1) and one unexposed death at d(2). At d(1) two subjects are under risk: one exposed, the other unexposed. Since the unexposed subject does not die at d(1) we get expected (d(1)) = (0/1) (1) cases among the one (1) exposed at d(1). At d(2) no exposed but one unexposed subject is under risk. Since the unexposed dies at d(2) this leads to expected(d(2)) = (1/1) (0) cases among the zero (0) exposed at d(2). Because one exposed death is observed at d(1) and zero exposed deaths at d(2), i.e., observed(d(1)) = 1 and observed(d(2)) = 0), it follows that

      e-YPLL = (1 - (0/1)1) YPLL(d(1)) + (0 - (1/1)0) YPLL(d(2))

      = YPLL(d(1)) = e-YLL.

Of course, using the second representation of e-YPLL given above one also gets

      e-YPLL = 1(d(2) - d(1)) - 0(0) = d(2) - d(1) = e-YLL.

Hence, e-YPLL measures e-YLL unbiasedly, given the elementary scenario and the exchangeability condition (together with other usual conditions necessary for study validity).

#### 2.2 Scenario with two pairs of twins

I have proven that e-YPLL is identical to e-YLL in an elementary setting of a cohort consisting of two exchangeable subjects given appropriate conditions. But does this hold if the cohort consists of two or more pairs of exchangeable twins? For didactic reasons I will study a cohort of two pairs of twins first. If we assume that exposure is never preventive, we are left with only two principal scenarios when investigating the situation with two pairs of twins. Indexing the pairs with a and b, the first principal scenario supposes the death times of the four subjects to be ordered as d_a_(1) ≤ d_a_(2) ≤ d_b_(1) ≤ d_b_(2). Obviously

    e-YLL = d_a_(2) - d_a_(1) + d_b_(2) - d_b_(1).

Now I will calculate the Years of Potential Life Lost under this scenario 1. For the sake of clarity, I add a second argument to the YPLL function denoting the pair under consideration (a or b). To simplify the notation of YPLL, I drop the letter d that is superfluous since we analyse a scenario without censoring (all subjects die under observation) (i.e., YPLL(a,1) = YPLL(d_a_(1))). The same simplified notation is used to denote observed and expected numbers of cases. Following Gardner and Sanborn 1990 [9] and using the ideal unexposed twins as the reference population the Years of Potential Life Lost at d_a_(1) are given as

    YPLL (a,1) = d_a_(2) - d_a_(1) + 0.5 (d_b_(2) - d_a_(2)).

This follows because all unexposed subjects survive at least to d_a_(1) and half of the unexposed subjects survive at least to d_a_(2). Analogous considerations yield

    YPLL (a,2) = d_b_(2) - d_a_(2)

    YPLL (b,1) = d_b_(2) - d_b_(1)

    YPLL (b,2) = 0.

The observed cases among the exposed are

    observed (a,1) = 1

    observed (b,1) = 1

and the expected, based on the unexposed, are given as

    expected(a,2) = (1/2)(1) = 0.5

    expected(b,2) = (1/1)(0) = 0.

Finally, this leads to

    e-YPLL = d_a_(2) - d_a_(1) + 0.5 (d_b_(2) - d_a_(2)) -

      - 0.5 (d_b_(2) - d_a_(2)) +

      + d_b_(2) - d_b_(1)

    = e-YLL.

Therefore, the equality of e-YPLL and e-YLL is proven for scenario 1. Next, I will investigate the other principal scenario. In contrast to scenario 1 the second principal scenario assumes the ordering d_a_(1) ≤ d_b_(1) ≤ d_a_(2) ≤ d_b_(2).

Given this scenario 2 we have

    e-YLL = d_a_(2) - d_a_(1) + d_b_(2) - d_b_(1)

    and

    YPLL(a,1) = d_a_(2) - d_a_(1) + 0.5(d_b_(2) - d_a_(2))

    YPLL(a,2) = d_b_(2) - d_a_(2)

    YPLL(b,1) = d_a_(2) - d_b_(1) + 0.5(d_b_(2) - d_a_(2))

    YPLL(b,2) = 0.

Moreover, it follows

    expected(a,2) = (1/2)0 = 0

    expected(b,2) = (1/1)0 = 0.

Therefore,

    e-YPLL = (1)YPLL(a,1) + (1)YPLL(b,1) - (0)YPLL(a,2) - (0)YPLL(b,2)

    = d_a_(2) - d_a_(1) + d_a_(2) - d_b_(1) + d_b_(2) - d_a_(2)

    = d_a_(2) - d_a_(1) + d_b_(2) - d_b_(1) = e-YLL.

If we assume exposure to be never preventive all possible configurations of the death times of both twins can be mapped onto these two principal scenarios by renaming the subjects accordingly. Thus e-YPLL = e-YLL is always valid in such an ideal study with two pairs of twins, although e-YPLL does not use the individual matching information.

It is important to note the following: the true excess Years of Life Lost can be calculated for each single pair as "observed cases times potential years of life lost" minus "expected cases times potential years of life lost" only in scenario 1. This calculation is obviously invalid under scenario 2. Only the total e-YPLL equals the total e-YLL in both scenarios. Thus, scenario 2 indicates that we have to be careful when a specified version of e-YPLL is applied to estimate the excess Years of Life Lost given specific conditions. Such an analysis is possible without bias if first, optimality criterion 2 is fulfilled as introduced in the last paragraph of Chapter 1, and second, if this individual matching information is used in the analysis. However, this kind of application is not justified if only criterion 1 holds. I will deal with this important issue of potential biases in Chapters 3 and 4.

#### 2.3 Scenario with n pairs of twins

A general proof of e-YPLL = e-YLL can be constructed using the principle of mathematical induction. Hence, this Chapter is a little bit technical. However, it may be skipped on first reading because it is not necessary to understand this proof to follow the arguments on potential biases developed in Chapters 3 and 4.

For the sake of clarity I introduce an additional functional argument denoting the number of pairs in the study: e-YLL(n) denotes the total excess Years of Life Lost due to exposure in a study with n pairs of twins. To start the inductive argument let us assume that e-YPLL = e-YLL has already been proven to be valid in all studies with n pairs of twins: e-YPLL(n) = e-YLL(n). This is the so called induction assumption. Now I add another pair of twins to the study and ask whether e-YPLL(n+1) = e-YLL(n+1). If I can prove this equality then it follows from the principle of mathematical induction that e-YPLL = e-YLL in all studies. This extension is true because I already have demonstrated that e-YPLL = e-YLL if n = 1 (start of induction).

To simplify the argument I now suppose the additional pair to have an unexposed twin with minimal survival among the unexposed n+1 subjects in the extended study. This simplication can always be assumed without any loss of generality for the following reason. If the added pair is not the one with the minimal death time among the unexposed I focus on a pair that fulfils that condition. Of course, such a pair always exists. Let us denote this pair with x and the death times of its twins with d_x_(1) and d_x_(2) accordingly. Regarding the other n pairs we know that e-YPLL(n) = e-YLL(n) due to the induction assumption. Hence it suffices to show that e-YPLL(n+1) = e-YLL(n+1) holds in the scenario with the added pair being pair x. This will prove the equation e-YPLL = e-YLL convincingly for all studies with n+1 pairs.

Since we assume throughout that exposure is never preventive we know that d_x_(1) ≤ d_x_(2). This means that the survival function of the exposed is the same in the extended study group with n+1 pairs as in the study group with n pairs, given we restrict the analysis to all exposed subjects with age at death greater than or equal to d_x_(2). Moreover, because d_x_(2) is minimal among the death times of all unexposed subjects the survival function of the unexposed is unchanged too. It follows that the Years of Potential Life Lost (i.e., conditional life expectancy) calculated from the unexposed reference population remains the same for all exposed subjects with age at death greater than or equal to d_x_(2). In addition, the expected number of cases among the exposed calculated from the unexposed remains unchanged. Therefore, the difference between e-YPLL(n+1) and e-YPLL(n) can only stem from events occurring before d_x_(2). In the next step I will calculate the change in e-YPLL caused by these events occurring before d_x_(2).

I assume that k of the n exposed in the study with n pairs may die before d_x_(2), 0 ≤ k ≤ n. Thus, there are n+1 - (k+1) = n - k exposed subjects of the extended study group under risk at d_x_(2), and it follows expected(x,2)=(n-k)/(n+1). The factor 1/(n+1) is to apply since the first event among the n+1 unexposed subjects occurs at d_x_(2). The amount of change in e-YPLL due to the additionally expected deaths at d_x_(2) is therefore given by -[(n-k)/(n+1)]YPLL(x,2). This is the first kind of contribution to the change in e-YPLL I have to consider. The second kind of contribution stems from the new exposed case dying at d_x_(1). This amount of change is simply the same as the Potential Years of Life Lost for this case: YPLL(x,1) = d_x_(2) - d_x_(1) + [n/(n+1)]YPLL(x,2) where n/(n+1) is the probability to survive d_x_(2) given the unexposed reference population. The third kind of contribution to the difference between e-YPLL(n+1) and e-YPLL(n) stems from the k exposed cases dying before d_x_(2). Their potential survival is shortened after introducing the new additional death at d_x_(2). Note that the reference-based probability to die at d_x_(2) is 1/(n+1) for each of these exposed cases. Therefore, the Years of Potential Life Lost is reduced for each of these cases by the amount [1/(n+1)]YPLL(x,2). This sums to [k/(n+1)]YPLL(x,2) for all k exposed cases dying before d_x_(2). Now I can calculate how e-YPLL changes when the pair x is added to the n pairs:

    e-YPLL(n+1) = e-YPLL(n) + d_x_(2) - d_x_(1) +

      + [n/(n+1) - (n-k)/(n+1) - k/(n+1)] YPLL(x,2)

      = e-YPLL(n) + d_x_(2) - d_x_(1)

      = e-YLL(n) + d_x_(2) - d_x_(1)

      = e-YLL(n+1).

The second to last equation follows from the induction assumption. The last equality is based on the obvious fact that the true excess Years of Life Lost increases by d_x_(2) - d_x_(1) if the pair x is added to the n pairs I started with. Hence, I have proven that e-YPLL equals e-YLL in all ideal studies consisting of pairs of exchangeable twins given that exposure is never preventive. Note that the measures are always calculated with respect to deaths from all causes. It is remarkable that the equality e-YPLL = e-YLL holds although e-YPLL does not make use of the individual matching information.

### 3. Non-identifiability of e-YLL conditional on age at death

**Figure 2:**
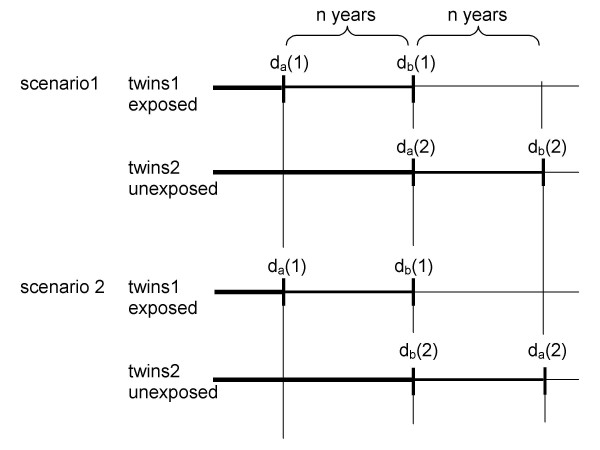
Two scenarios of different exposure effects in two pairs of twins. The pairs are indexed by a and b. As before, d_a_(1), d_b_(1) are death times of the exposed and d_a_(2), d_b_(2) are death times of the unexposed twins. Scenario 1 assumes causal effects of the same degree in both pairs, d_a_(2) - d_a_(1) = d_b_(2) - d_b_(1) = n years. Scenario 2 supposes no effect in pair b, d_b_(1) - d_b_(2) = 0 years, and a large one in pair a, d_a_(1) - d_a_(2) = 2n years.

I have shown in Chapter 2 that the total e-YLL of the whole study group can be measured accurately by the total e-YPLL provided the assumptions of an ideal study hold including optimality criterion 2 (cf. last paragraph of Chapter 1) and provided the analysed response is death from all causes. However, this identity of e-YLL and e-YPLL no longer holds if I am interested in e-YLL conditional on age at death. To see why consider the scenarios illustrated in Figure 2.

In both scenarios (Fig. 2) the total e-YLL's are identical and thus, are also the total e-YPLL's. However, the e-YLL conditional on age at death varies with the scenarios: the Years of Life Lost due to exposure at d_a_(1) are smaller in scenario 1 than in scenario 2 whereas it is vice versa at d_b_(1). Irrespective of the scenario, the e-YPLL are always calculated in the same way. Therefore, we get in both scenarios the same e-YPLL at d_a_(1) as well as the same e-YPLL at d_b_(1). Note that the calculation of e-YPLL does not make any use of the individual matching information.

Hence, the true excess Years of Life Lost conditional on age at death are not identifiable without having access to a perfect control twin or without supposing a specific mechanism for how exposure causes death. Note that criterion 1 alone – as defined in the last paragraph of Chapter 1 and hopefully fulfilled in usual cohort data – does not render the excess Years of Life Lost stratified on age at death identifiable. Consequently, estimates based on e-YPLL conditional on age at death are potentially biased in all usual settings.

Next, I analyse a theoretical birth cohort of 100,000 men to demonstrate this potential bias by a realistic life table example (Table 1, see Additional file 1). I assume the death rates of the male US population as tabulated in BEIR IV 1988 [25] as reference rates. The example is restricted to the unexposed men surviving at least to an age of 30 years.

Life expectation at death is calculated from the size of the unexposed population and the number of unexposed deaths, presuming that all deaths occur at the midpoint of age categories. Numbers of exposed deaths include a certain advancement of age at death without changing the totals. Excess deaths are calculated as the difference between the number of exposed deaths and the expected deaths among the exposed. The latter is determined by multiplying the size of the exposed population by the fraction of deaths among the unexposed population conditional on age. Finally, e-YPLL is derived according to Park et al. 2002 [2] by multiplying the number of excess deaths by the life expectation at death in each age category (see Chapter 2.1 for details). The total number of excess Years of Life Lost due to exposure sums up to 255,094 years.

Table 2 (see Additional file 2) describes the impact of two different causal exposure-response mechanisms, both compatible with the data presented in Table 1 (see Additional file 1). Mechanism 1 assumes two different response types of subjects. One type is immune and does not react under exposure in comparison to no exposure (no advancement of age at death). The other type shows an adverse effect of exposure because his death is advanced by five years. In contrast, mechanism 2 supposes three different reaction types: an immune one and two types adversely affected by exposure. The affected types differ in their degree of response (advancement by five years or ten years). Tables 1 and 2 are based on a spreadsheet that is supplied as an additional data file (see Additional file 4).

**Figure 3:**
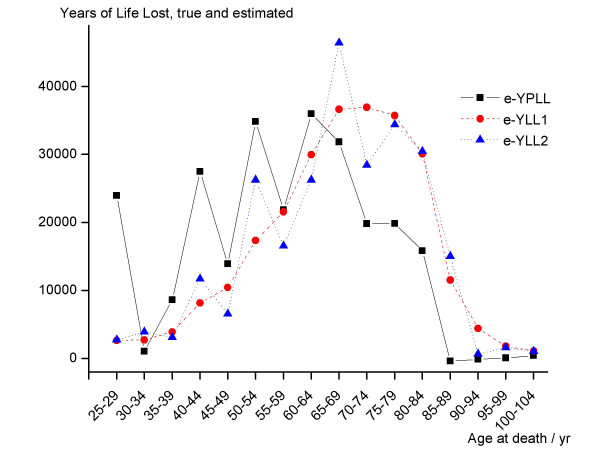
Excess Years of Potential Life Lost e-YPLL and true excess Years of Life Lost e-YLL1 and e-YLL2, assuming two different causal mechanisms, conditional on age at death. Basic data, exposure impact, and mechanism producing e-YPLL, e-YLL1, and e-YLL2 are given in Table 1 and Table 2. Lost Years, true and estimated, sum up to 255,094 years always.

Note that the total numbers of deaths, unexposed and exposed, as well as the total excess Years of Life Lost are identical in both sub-tables of Table 2 (see Additional file 2) although the sub-tables reveal a different causal impact of the varied mechanisms. Note further that the distribution of unexposed deaths as well as the distribution of exposed deaths are constant across both sub-tables and Table 1 (see Additional file 1). Moreover, the total e-YLL calculated in the sub-tables of Table 2 (see Additional file 2) agrees with the total e-YPLL derived in Table 1 (see Additional file 1). But obviously, the excess Years of Life Lost conditional on age differ between mechanism 1 and 2, and moreover, diverge from the conditional excess Years of Potential Life Lost calculated in Table 1 (see Additional file 1). Figure 3 contrasts the distributions.

The excess Years of Potential Life Lost differ remarkably from the true excess Years of Life Lost. The difference depends on the chosen mechanism. In this example e-YPLL overestimates the true excess Years of Life Lost up to an age of about 65 years and underestimates the impact at higher ages. The zigzag increase of e-YPLL may reflect a real phenomenon since mechanism 2 produces a similar structure. However, the somewhat surprising zigzag increase of e-YPLL may be totally artificial because mechanism 1 causes a nearly smooth incline. Thus, it is not justified to interpret the form of the increase of e-YPLL across age as a true phenomenon without assuming untestable mechanistic assumptions about how exposure causes death. Note that such naive interpretations can be grossly misleading.

Hence, as illustrated by this example, the true excess Years of Life Lost conditional on age at death cannot be identified from cohort data like that presented in Table 1 alone (survival curves, see Additional file 1). The estimator e-YPLL conditional on age is shown to be potentially biased even if optimality criterion 2 (as defined in the last paragraph of Chapter 1) is fulfilled. The reason is that e-YPLL conditional on age does not make use of the relevant individual matching information.

### 4. Non-identifiability of Years of Life Lost due to a specific cause of death

**Figure 4:**
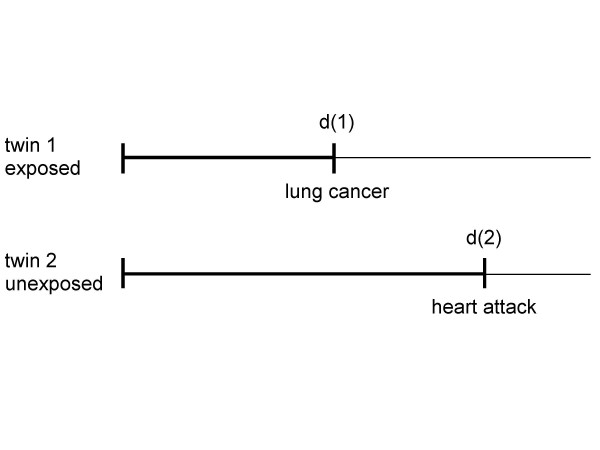
Two exchangeable twins 1 and 2. The exposed twin died from lung cancer at age d(1), the unexposed twin 2 died from heart attack at d(2) > d(1).

The equality of total e-YPLL and total e-YLL was proven for deaths from all causes given the assumptions mentioned in Chapter 2. However, such an equality does not hold for a specific cause of death like lung cancer. To see why, consider the simple scenario presented in Figure 4.

Figure 4 illustrates an elementary gold standard cohort study performed with two twins, one exposed and the other unexposed. Since the subjects are assumed to be exchangeable, exposure caused a premature death with an overall e-YLL = d(2) - d(1). However, if we are interested in the exposure impact on age at death for lung cancer we are left with an identification problem. Obviously, if twin 2 had died from lung cancer also the causal effect on age at death from lung cancer would have been d(2) - d(1). But in case of the scenario illustrated in Figure 4, one can only conclude that the effect on age at death from lung cancer must be at least d(2) - d(1). Therefore, as a simple but important consequence, the causal effect on age at death from lung cancer is not identifiable even in an optimal study comprising exchangeable twins as long as competing causes of death exist. Since this causal effect cannot be identified even in an optimal study (i.e., fulfilling condition 2 defined in the last paragraph of Chapter 1) it does not make sense to estimate it without assuming untestable mechanistic assumptions about how exposure causes death from lung cancer.

Note, however, that this causal effect is not the e-YLL due to exposure and manifested by lung cancer, e-YLL(lung cancer) for short. In contrast to the causal effect, e-YLL(lung cancer) comprises exactly that part of e-YLL from all causes of death that is due to the occurrence of lung cancer deaths among the exposed. In the example (Figure 4) these e-YLL(lung cancer) are d(2) - d(1) which is the exposure effect on the age at death from all causes, e-YLL(overall) for short. The reason ist that there are no other causes of death among the exposed besides lung cancer. Whereas the causal effect of exposure on age at death from lung cancer cannot be identified in an optimal study with pairs of twins fulfilling optimality criterion 2 (defined in the last paragraph of Chapter 1) without adopting further untestable assumptions we may ask whether at least e-YLL(lung cancer) can be estimated unbiasedly assuming criterion 2. To give an answer we must investigate the situation in more detail.

**Figure 5:**
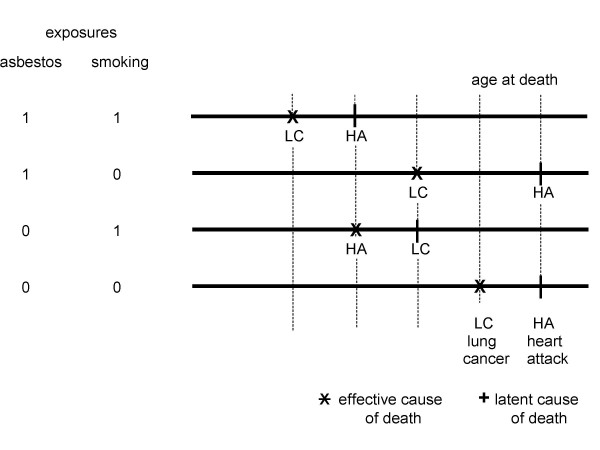
Effective and latent causes of death in a quadruplet of exchangeable subjects. Each subject is supposed to have been allocated to a different combination of two binary exposures, illustrated here by asbestos and smoking. Without any exposure and as well as after asbestos exposure the subjects are assumed to have died from lung cancer, LC (effective cause). Lung cancer was latent in the smoker not exposed to asbestos while heart attack, HA, became the effective cause in this situation. Asbestos exposure had no causal effect on age at death from heart attack but from lung cancer. Smoking affected age at death from heart attack and lung cancer, the latter to the same amount as asbestos did. Under joint exposure a synergistic effect on age at death from lung cancer is assumed, but not for heart attack.

The concept presented supposes that each subject has a set of hypothetical (deterministic) death times for each combination of level of exposure and cause of death. Just one of these death times is effective, the others are latent. For the exposed twin 1 lung cancer is effective as a cause of death and heart attack is latent. It is vice versa for the unexposed twin 2. Both causes of death compete within each twin for the effective position. Of course, the result of this competition depends on the exposure conditions applied. This result motivates to introduce a second (competing) exposure into the scenario, in which I deal with two competing responses (lung cancer and heart attack). Figure 5 expands on this concept accordingly.

In Figure 5 both exposures are assumed to be never preventive for all endpoints of interest (LC = lung cancer, HA = heart attack). However, when contrasting the two situations without asbestos exposure smoking obviously leads to a decrease in the number of lung cancer deaths, and correspondingly, asbestos exposure causes a decrease in the number of deaths from heart attack given smoking. This spuriously preventive effects of both exposures are an outcome of the competing causes of death structure. In the following, I prove that this structure leads to a potential bias of e-YPLL(lung cancer). Note that the misleading impression of a preventive effect is produced by relying on a risk or rate statistic only. As I emphasized in the Introduction section, these statistics count accurately how many responses of what kind occurred ("whether"), but unfortunately, mainly ignore the specific causal structure in time ("when").

**Figure 6:**
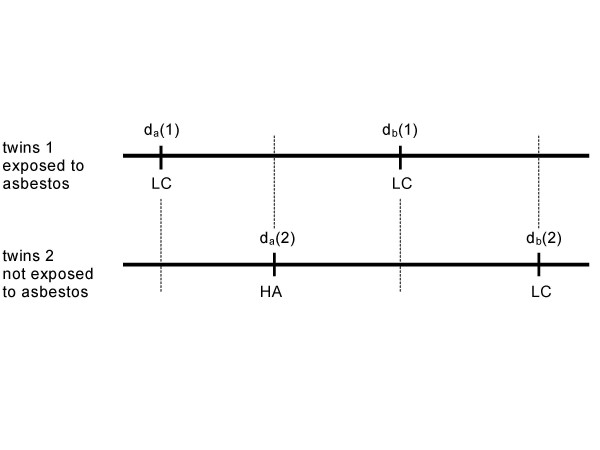
The effective data of Figure 5 analysed in an ideal cohort study consisting of two pairs of twins which are supposed to form a quadruplet of exchangeable subjects, cmp Figures 1 and 2. The pairs are indexed by a and b: the twins in pair a are assumed to have been smokers, those in pair b non-smokers; d_a_(1), d_b_(1) are death times of the asbestos exposed and d_a_(2), d_b_(2) are death times of the twins not exposed to asbestos. The effective cause of death, lung cancer LC or heart attack HA, are denoted at age at death, accordingly.

From Figure 6 I conclude

    e-YLL(overall) = e-YLL(lung cancer) = e-YPLL(overall) =

    d_a_(2) - d_a_(1) + d_b_(2) - d_b_(1).

The first equation holds because the only cause of death among all asbestos exposed is lung cancer and the second because I have shown that e-YLL(overall) = e-YPLL(overall) is true in an ideal study given the aforementioned assumptions. The third equation follows from a simple evaluation of e-YLL(overall).

Next, I calculate e-YPLL(lung cancer) according to Park et al. 2002 [2]:

    e-YPLL(lung cancer) = Σ (observed_lungcancer_(t) - expected_lungcancer_(t)) YPLL(t).

The only change in comparison to the formula of e-YPLL introduced in Chapter 2.1 is the specification of the observed and expected cases. The important point here is that the procedure, as defined by Park et al. 2002, only uses information at age at death from lung cancer. For the YPLL-terms involved I get

    YPLL (a,1) = d_a_(2) - d_a_(1) + 0.5 (d_b_(2) - d_a_(2))

    YPLL (b,1) = d_b_(2) - d_b_(1),

from which I derive

    e-YPLL(lung cancer) = d_a_(2) - d_a_(1) + 0.5 (d_b_(2) - d_a_(2)) +

      + d_b_(2) - d_b_(1)

      = e-YLL(lung cancer) + 0.5 (d_b_(2) - d_a_(2)),

since no expected lung cancer deaths are to be subtracted.

Hence, e-YPLL(lung cancer) is biased upward by 0.5 (d_b_(2) - d_a_(2)) which is half of the causal effect of smoking on age at death from all causes (overall) according to Figure 5. This overestimate is due to the fact that no lung cancer deaths are expected to occur among the exposed during follow-up. This empirical shortcoming of e-YPLL(lung cancer) could only be overcome in general if we were able to identify the matched partner of the lung cancer case not dying from lung cancer and if we use this information in the analysis. Since this is impossible when relying on usual cohort data (survival curves) I have proven the non-identifiability of the excess Years of Lost Life due to exposure for a specific cause of death, given the natural situation of competing causes for death and usual cohort data. I demonstrate the potential bias of e-YPLL(lung cancer) by a life table example additionally (Table 3, see Additional file 3). This table relies on a spreadsheet which is also supplied (see Additional file 5).

Table 3 (see Additional file 3) uses the same basic data as Table 2 (see Additional file 2) to describe the unexposed population but is extended by an additional column presenting the number of lung cancer deaths by age. The impact of exposure on lung cancer death is defined by an advancement of age at death by five years in all situations. Thus, factual (effective cause) age at death as well as hypothetical (latent cause) age at death is assumed to occur five years earlier under exposure. Furthermore, it is supposed that the advancement of latent lung cancer deaths leads to an excess of 50% among unexposed lung cancer deaths in each age category, if exposed. This advancement means that 3367 (= 10102 - 6735) subjects died from lung cancer after exposure who are assumed to have died from other causes if unexposed. A mixture of advancements greater or equal than 0 years, differing in amount between other causes of death than lung cancer, is assumed to produce the distribution of the number of deaths from all causes among the exposed. Hence, the overall excess Years of Life Lost for lung cancer are less than the number of exposed lung cancer deaths times five years (i.e., 50,509 years). This number is an upper bound because the additional lung cancer deaths occurring under exposure are related to a loss of life in the individual of at most five years while the cause of death changes from, say, heart attack to lung cancer.

Applying the procedure proposed by Park et al. 2002 [2] I calculate a total e-YPLL(lung cancer) of 86,373 years. Thus, the estimate is biased upward by more than 35,000 years which corresponds to a relative bias of at least 70%. In addition, the life table analysis demonstrates that the e-YPLL method is also biased when used to estimate the causal effect of exposure on age at death from lung cancer among the exposed lung cancer cases, which exactly amounts to 50,509 years. Moreover, the causal effect of exposure on the lung cancer death times among all subjects was defined to be 5(92,219) years = 476,095 years, again clearly different from e-YPLL(lung cancer) = 86,373 years.

## 5. Discussion

I have shown that the total excess Years of Life Lost (e-YLL) for death from all causes can be accurately determined by calculating the corresponding excess Years of Potential Life Lost (e-YPLL) provided conditions hold that are also often cited for general study validity. However, the equality of e-YLL and e-YPLL does not hold in general under certain conditions of interest because under these certain conditions we need to assure criterion 2: each exposed subject has to have an ideal unexposed control partner so that the effect of exposure can be measured on the individual level (see also the last paragraph of Chapter 1). The equality also requires that this information about individual matching is used in the analysis. I pointed out that the e-YPLL conditional on age at death are potentially biased as they were published in some analyses (see e. g., Figure 1 in Park et al. 2002 [2]). Furthermore, the excess Years of Potential Life Lost calculated for a specific cause of death are potentially biased also. Hence, estimates of Years of Life Lost presented for chronic diseases like lung cancer (see for example the detailed lung cancer Poisson model analysis by Park et al. 2002 [2]) are unjustified without referring to untestable assumptions about the causal mechanism involved.

I should add, just for the sake of clarity, that in all my examples presented that cast doubt on the validity of e-YPLL, exposure was always assumed to be never preventive. Therefore, preventive effects of exposure are not the reason for these problems. The deeper reason of these validity problems of e-YPLL is a non-identifiability of excess Years of Life Lost due to exposure in an ideal but unmatched cohort study. Note further that the calculation of e-YPLL will ignore this important matching information even if such data are available. This structural problem of non-identifiability was already clearly described, analysed and discussed on an abstract level by Robins and Greenland 1991 [1], even under the generalized scenario of stochastic responses on the individual level. For further theoretical discussions of the problems involved and for an investigation into untestable assumptions that render identification possible, I therefore like to refer to this important publication. I simply remark here that mechanisms 1 and 2 (Table 2, see Additional file 2) do not preserve ranks.

It is important to note that the validity problems of e-YPLL cannot be resolved by applying the rare disease assumption or by restricting the discussion to a situation with a constant relative risk across age. Rather, the problems are structural ones. For example, if the additional lung cancer cases under exposure are calculated in Table 3 (see Additional file 3) by applying an increasing factor with age at death instead of using the constant value of 1.5, the SMR's for lung cancer can be kept almost constant across age. However, e-YPLL still overestimates the true excess Years of Life Lost due to lung cancer by a considerable amount. A substantial upward bias, nearly independent of the increasing factor, can be demonstrated by simulations that assume an isolated and homogeneous detrimental effect of exposure on lung cancer. This describes a scenario where other endpoints are only changed due to exposure because they are competing with lung cancer. Again using the data from BEIR IV 1988 [25] (Table 3, see Additional file 3) I find an upward relative bias of at least 30% if the additional lung cancer deaths (that take over the effective position from competing causes if exposed) amount to about half of the lung cancer deaths shifted to a younger age at death (under exposure) without changing the endpoint. The examples I studied show that the relative bias is at least 70% if both groups are of the same size.

In agreement with the direction of bias indicated by the abstract quadruplet analyses of total excess Years of Life Lost for lung cancer (Figures 5 and 6) I show an upward distortion of e-YPLL(lung cancer) in the life table example (Table 3, see Additional file 3). This upward bias is due to an inappropriate mixture of life expectancy at age at death from lung cancer, based on data from all later deaths, with lung cancer excess deaths only, while ignoring the corresponding excess deaths from competing causes among the non-exposed that are produced as a side-effect of the causative exposure (against the interpretation of Park et al. 2003 [15]). However, total e-YPLL for a specific cause of death can be used to assess total e-YLL for this cause unbiasedly if despite potentially competing causes no change of the cause of death occurred within the whole cohort after a different exposure level of interest had been allocated counterfactually. Unfortunately, this assumption is not provable. When excess cases of the cause of death of interest are observed among the exposed – a situation quite natural in occupational cohort studies – this assumption definitely does not hold.

My analysis reported so far on problems with ideal studies with complete follow-up. Obviously, if a constant end of follow-up is introduced into the scenarios of Figure 5 and 6 so that the number of lung cancer deaths are affected, the calculated e-YPLL can change whereas the true e-YLL always remain constant. Hence, the degree of bias depends additionally on the censoring structure even if left censoring is totally uninformative. This dependence means that censoring is not taken into account adequately by the e-YPLL method when applied to specific endpoints.

One important point to discuss is whether the constructed life table examples are realistic ones in comparison to the data and results published in Park et al. 2002 [2]. All tables presented here are based on the mortality experience of the male US population as published by BEIR IV 1988 [25], Table 2A-10, p. 139. Since the paper of Park et al. 2002 [2] analysed the mortality of white US uranium miners, hired during 1950 - 1963 and followed until 1990, the choice of this reference population appears to be appropriate.

Park et al. 2002 [2] described the total excess Years of Life Lost per cohort member as 3.1 years (Table IV, page 6) and an effect of similar magnitude was chosen here: 2.7 years per cohort member (n = 95,219) according to Tables 1 or 2 (see Additional file 1 and 2), and 3.3 years per cohort member according to Table 3 (see Additional file 3). In Park et al. 2002 [2] the lung cancer SMR dropped from 7.6, age period 30–34, to 5.4, age band 45–49, and even further to SMR = 2.6, age period 65–69 (Table III, page 5). The example given in Table 3 (see Additional file 3) tries to mimic this downward trend starting with 7.0 in age category 30–34, then decreasing to 3.0 in age band 45–49 and to 1.7, age period 65–69. Thus, the overall downward trend is somewhat more pronounced here than in Park et al. 2002 [2]. However, I try to simulate a trend of SMR's across age that is quite similar to the relative decrease observed within the main body of data in Park et al. 2002 [2] (age bands 65–69/45–49): 2.6 / 5.4 = 48% (Table III, Park et al. 2002 [2]) and 1.7 / 3.0 = 57% (Table 3 of this paper, see Additional file 3).

The overall lung cancer SMR can be calculated from Table 3 (see Additional file 3) as 1.74, less than the value of 3.8 observed by Park et al. 2002 [2] (Table III, page 5). A lower SMR was chosen here to avoid an exaggeration of potential biases due to overstating the effect of exposure in the constructed example. Accordingly, the e-YPLL for lung cancer was presented as 1.47 years per cohort member by Park et al. 2002 [2] (Table IV, p. 6) but only 0.91 years per cohort member (n = 95,219) can be calculated from Table 3 of this paper (see Additional file 3).

Hence, the life table examples presented in Tables 1, 2 and 3 (see Additional file 1, 2 and 3) are realistic ones and the magnitude of potential biases identified appears to be defensible. The potential biases derived are fairly extreme: the true total Years of Life Lost are overstated by 100% (mechanism 1) or 33% (mechanism 2) in age band 50–54 but understated by more than 40% in age period 75–79 (Figure 3 and Tables 1 and 2, see Additional file 1 and 2). Moreover, a relative upward bias of more than 70% is demonstrated for the e-YPLL related to lung cancer (Table 3, see Additional file 3). These findings render the central results published by Park et al. 2002 [2] scientifically unreliable (i.e., the age-specific analysis of excess Years of Life Lost due to exposure as well as all results published on excess Years of Life Lost in relation to lung cancer). Note that the latter was the main topic of Park et al. 2002 [2].

Due to its definition (Breslow and Day 1987 [24]) the average lung cancer SMR is calculated as observed / expected = exposed / (exposed - excess) which leads to 10,101.74 / (10,101.74 - 4,286.88) = 1.74 (Table 3, see Additional file 3). For the sake of clarity I should note that this value does not coincide with the average causal case ratio (exposed / unexposed = 10,101.74 / 6,734.49 = 1.5). The discrepancy is due to the fact that the construction of all life tables in this paper strictly follows counterfactual principles: the elevated death rates in the exposed population affects the person-years yielding an expected number of lung cancer cases smaller than the true observed number of lung cancer cases in the unexposed population (cp. Keiding and Vaeth 1986 [26], Greenland 1996 [27]).

Statistical measures that focus on the advancement of disease onset are assumed to be helpful in communicating risk factor impact on disease (e.g., Peto 1980 [28]). Fischhoff et al. 1993 [29], Jardine and Hrudey 1997 [30] as well as Yamagishi 1997 [31] pinpointed problems in risk perception and communication when frequency statistics are solely presented as descriptors of health effect. Weinstein et al. 1996 [32] demonstrated additionally a considerable effect of framing risk levels on the perception of risk.

Statistics conveying information about the advancement of disease onset are therefore helpful in exposure impact analysis and especially worthwhile in exposure impact communication. However, attention should be drawn to the difficulties involved and that epidemiologists should always be aware of the conceptual limits of the Years of Potential Life Lost method when applying it as a regular tool in cohort analysis.

Aside from Years of Life Lost, other approaches are available to convey information on the impact of a risk factor on the onset of a disease and may thus facilitate communication of epidemiological findings. One such concept is the risk and rate advancement period (RAP) introduced by Brenner et al. 1993 [33] which could easily be calculated from standard software output. Risk and rate advancement periods are the time periods by which the risk or rate of disease is advanced among exposed subjects given a disease-free survival to some baseline age. An application of RAPs in assessing the impact of risk factors on myocardial infarction was published by Liese et al. 2000 [34]. However, the RAP statistic cannot be applied if disease rates do not increase strictly with age. Another approach describing the exposure impact on disease onset, complementary to RAP, was developed by Boshuizen and Greenland 1997 [35]. The authors estimated the time shift in average age at first occurrence of disease due to exposure as a measure of disease advancement. Although especially valuable when the background incidence of the disease is high, a technical drawback of this procedure stems from the necessity to correct regression model likelihoods for left truncation, an option that is not offered in standard statistical packages. Of course, none of these procedures can be applied sensibly if, according to study design, cases and controls or exposed and unexposed were matched on age. A rigorous causal approach in estimating the shrinkage or extension of time to an event, like lung cancer death, due to exposure was developed by Jamie Robins and is called G-estimation (for a brief overview see Rothman and Greenland 1998 [5], p. 424, 425). Since this method has a profound logical basis and can be applied even in complicated longitudinal scenarios with interrelated time-dependent covariates, and in addition, is programmable in standard software like SAS or STATA it should become a regular tool in cohort analysis, in particular when Years of Life Lost are to be estimated.

The main conclusions about non-identifiability of e-YLL are derived as an application of a causal theory based on counterfactuals (for a review see Greenland 2000 [36]). Some authors seem to believe that this approach has no clear logical and philosophical backing, judging Greenland's reasoning as purely academic (Armstrong and Thieriault 1996 [37]) or Robin's counterfactual based methods like G-estimation as not applicable in occupational epidemiology (Steenland et al. 1996 [38]). The arguments and examples given in this article prove the applicability and practical relevance of the counterfactual approach in occupational epidemiology. However, this does not help to eliminate a more fundamental philosophical resistance I experienced in a number of controversial discussions (e. g., with epidemiologist from NIOSH, Cincinnati). Thus, some explanation of the philosophical background of counterfactual reasoning should be given here.

The ideas of counterfactual reasoning can at least be traced back to philosophers in the eighteenth and nineteenth centuries, like Hume and Mill. Even the oldest clearly structured theory of causality, developed by Aristotle, has some similarities to counterfactual reasoning due to its manipulative four-causes concept (Vorländer 1979 [39]). In its most simple form, the counterfactual approach assumes the existence of potentially different hypothetical response variables for the same subject depending on differently assumed exposure conditions. At most one of these conditions and responses can become true, the others remain hypothetical, so counterfactual. A causal comparison is understood as a comparison of the responses due to different exposure conditions within the same subject. Indeed, this is the rigorous version of ideal twins I have used throughout to derive statements about causality (see Figures 1, 2, 4, 5, 6). In their famous statistical textbook, Cox and Oakes 1985 [40], p. 64 implicitly used this approach to define the stronger version of the accelerated failure time model: "any individual having survival time t under z = 0 would have survival time t/Ψ under z = 1". The counterfactual nature of this statement is reflected by the tense used (conditional II). Note that modern physics also uses counterfactual reasoning, expressed in conditional II, to define causes in the framework of the general theory of relativity: "We say that an event, let us call A, is in part the cause of another event, B, if A was necessary for B to occur. If A had not occurred, B could not have. In this case I can say that A was a contributing cause of the event B" (Smolin 2001 [41]). And note further that also everyday causality is often expressed by statements in conditional II (i.e., by statements that describe situations that contrary to fact did not occur – "If the train had not stopped for such a long time in front of the railway station I would have reached the connecting train"). A detailed philosophical discussion of the counterfactual causal model is given in Lewis 1973 [42].

Some authors resist a counterfactual approach and argue against the speculative and metaphysical background of counterfactual worlds (Dawid 2000 [43]): How can one object have two different contradictory properties like being exposed (factually) and simultaneously not being exposed (counterfactually)? In addition, the units of observation are assumed to exist in different states simultaneously in the counterfactual world if the properties have more than two distinct values. This duality appears to be even more puzzling. However, exactly such weird statements are made by quantum mechanics about objects like electrons or Fulleren molecules which are part of the real world we live in (Mittelstaedt 1972 [44], Feynman 1985 [45], Zeilinger 2003 [46]). It is important to understand that this superposition principle ("superposing" contradicting properties simultaneously on the same object) is no marginal phenomenon but lies at the center of modern physics (Dirac 1967 [47]).

Note further that the mathematically consistent analytical treatment of causal questions by counterfactual theory is obviously related to the so called multiverse approach (Everett 1957 [48]). The latter is a suggestive ontological interpretation of abstract Hilbert space theory that was introduced into modern physics to represent quantum states (Weberruβ 1998 [49], Penrose 1994 [50], Deutsch 1997 [51]). A new mathematical approach was necessary because – due to the superposition principle – quantum states are much richer than classical states and therefore fall beyond the grasp of Newtonion and Einsteinian terminology and theory (Hughes 1999 [52]). Critics of counterfactual logic, like Dawid 2000 [43], overlook this deep connection to quantum mechanics that renders the approach empirically plausible and mathematically as consistent as modern physics. In other words, a fundamental philosophical attack of counterfactual reasoning leads inevitably into an attack of quantum physics, which has survived successfully all criticism raised, empirically and theoretically, during the last century (Feynman 1985 [45], Hawking 1996 [53], 2001 [54]). In a discussion of the so called null measurements in quantum mechanics Roger Penrose described the link of quantum mechanics to counterfactuals as follows: "It is quite extraordinary that quantum mechanics enables you to test whether something might have happened but didn't happen. It tests what philosophers call counterfactuals. It is remarkable that quantum mechanics allows real effects to result from counterfactuals!" (Penrose 2000 [55], p. 67).

In addition, this link to quantum mechanics disproves the repeatedly made statement by Dawid (cp. the discussion following Maldonado and Greenland 2002 [23]) that counterfactual reasoning were subject to a deterministic or 'fatal' world view. On the contrary, quantum mechanics is the realm of pure chance and statistics: counterfactuals are therefore clearly consistent with indeterminism and stochastic principles. Finally, critics of counterfactual logic, like Dawid 2000 [43], have not presented an appealing and logically consistent alternate mathematical theory of causality.

Whereas the counterfactual approach can help to clarify terminology and substance of causal relations, it points simultaneously at some ambiguities when discussing competing causes of death. In this scenario, it is unclear what kind of action should be taken to cause a suppression of competing risks (Greenland 2002 [56]). Hence, the logical and causal background of the example presented with competing causes of death (Figure 4, 5, 6 and Table 3, see Additional file 3) appears to need further elaboration.

## Conclusion

In conclusion, the excess Years of Potential Life Lost estimates the excess Years of Life Lost due to exposure unbiasedly if we are interested in a) death from all causes and b) total excess Years of Life Lost summed up across the whole cohort. However, the method of calculating excess Years of Potential Life Lost due to exposure is potentially biased if it is applied 1) to estimate the impact of exposure on specific causes of death, like lung cancer, in the presence of competing causes or 2) to estimate the impact of exposure (overall or cause specific) conditional on age at death. These potential biases can be rather severe in published analyses (e.g., Park et al. 2002 [2]). Rigorous causal thinking (Greenland et al. 1999a [57], Greenland et al. 1999b [58]) can help to identify and avoid such empirical shortcomings. Fortunately, as an important outcome of these causal considerations, methods are available (Robins 1997 [22]) and readily applicable in occupational epidemiology (Witteman et al. 1998 [59]) to estimate the cause-specific reduction in life span due to occupational exposures unbiasedly given a specified failure time model and the assumption of no unmeasured confounders. These methods are valid even under the complicated but often realistic conditions of dependent censoring, survivor biases and intermediate confounding (Keiding et al. 1999 [60], Morfeld et al. 2002 [61]).

## Competing Interests

The author(s) declares that he has no competing interests.

## Endnote 1

The following example is based on the counterfactual framework presented in Chapter 1. It demonstrates that neither relative risks nor relative rates can be used in general to estimate the probability of causation unbiasedly. In particular, I show that an estimate of the attributable hazard derived from a Cox model (Breslow and Day 1987 [24]) fails to describe the causally attributable fraction among the exposed cases. The example demonstrates that this shortcoming is due to the fact that an advanced onset of disease is not reflected completely by risk or rate statistics. As in Chapter 1, a binary exposure indicator is assumed for simplicity in the following.

The cohort is supposed to comprise four subjects: A, A's twin, B, and B's twin. In addition, it is assumed that the cohort is followed up in mortality until the fixed censoring date t_end_>0. The time scale can be chosen arbitrarily as age, calendar time or time since start of follow-up without affecting the following arguments. I assume that the exposed subject A may experience the event (death) during the follow-up period at t_1 _years (t_1 _> 0), if unexposed counterfactually (A's twin) at t_2 _> t_1_, t_2 _< t_end_. No event may occur in subject B and his/her twin during follow-up (i.e., neither under exposure nor when unexposed). Thus, among the two exposed subjects, A and B, only one case occurs and this case is causally affected by exposure since t_1 _< t_2_. It follows that the probability of causation (i.e., the percentage of exposed cases occurring during follow-up which are causally affected by exposure) is exactly 100%.

In contrast, the incidence proportion (Rothman and Greenland 1998 [5]) is 0.5 among both, the exposed and unexposed, leading to a relative risk of 1 and an attributable risk among the exposed of 0. Hence, the attributable risk calculated from incidence proportions fails to reflect the causal impact of exposure on subject A's event time (true probability of causation among the exposed = 100% >> attributable risk among the exposed calculated from incidence proportions = 0%). I'd like to emphasize that the reason for this shortcoming is the fact that relative risks reflect excess cases only but no advanced cases.

Note that a change from risks to rates does not overcome the problem. The rate among the exposed is 1/(t_1 _+ t_end_) and among the unexposed is 1/(t_2 _+ t_end_). Consequently, the rate ratio is (t_2 _+ t_end_)/(t_1 _+ t_end_) > 1 because t_2 _> t_1_. The attributable rate among the exposed can be determined as (rate ratio -1) / rate ratio = 1 - 1/rate ratio. Thus, I conclude 0 < 1 - (t_1 _+ t_end_)/(t_2 _+ t_end_) < 1, again proving a systematic underestimate of the true probability of causation among the exposed by the attributable rate ratio. Note that the rare disease assumption is of no help. Assuming n exposed subjects (n>>1) that neither react nor do their n unexposed twins, we get an attributable rate among the exposed of 1 - (t_1 _+ (n)(t_end_)) / (t_2 _+ (n)(t_end_)) < 1. If n approaches infinity the attributable rate among the exposed decreases to zero whereas the true probability of causation remains always constant at 100%. Hence, the discrepancy is even sharpened under the rare disease assumption.

Last, I try to escape these problems by performing a Cox analysis. Due to its construction, the whole cohort (exposed and unexposed) comprises 4 subjects and 2 risk sets. One set is generated by the exposed case (A), the other set by the unexposed (A's twin). Therefore, a Cox analysis of the cohort yields the following.

Assuming a relative hazard (rate) in the Cox model of

    λ / λ_0 _= exp (β exposure), exposure = binary exposure indicator

we get the partial likelihood

    

which is maximized at b = ln 2/2.

Therefore, the estimated hazard ratio is

    exp (b) = √ 2

yielding an estimated attributable hazard among the exposed of

1 - (√2)^-1 ^< 1 = probability of causation.

Note that the rare disease assumption is again of no help to overcome this discrepancy because the estimated attributable hazard among the exposed approaches 0 when the number of controls is rising indefinitely.

Greenland 1999 [6] emphasizes correctly that this methodological error (i.e., using attributable risks or rates among the exposed to measure the probability of causation) has already become a social problem. Compensation of workers who developed a disease after occupational exposure is often decided by impact measures based on this erroneous identification of probability of causation and attributable risk or rate among the exposed. Usually a threshold value of 50% is chosen in Germany, which corresponds to the so called doubling of risks (i.e., a relative risk or rate of 2). Thus, compensation is usually withheld if the attributable risk or rate is below 50% (Morfeld and Piekarski 2001 [7]). Similar procedures are applied in other countries (Greenland 1999 [6], Armstrong and Theriault 1996 [37]). However, as demonstrated in this endnote, such an argument based on the attributable risk or rate is not justified. Discussions have started about how the discrepancy between the calculated attributable risk among the exposed and the intended probability of causation can be accounted for in amended compensation schemes in Germany (Morfeld und Piekarski 2001 [7]). These amended compensation schemes should reflect the advancement of disease onset due to exposure more accurately than the conventional ones.

## Supplementary Material

Additional File 1 Life table analysis with calculation of excess Years of Potential Life Lost e-YPLL according to Park et al. 2002. Basic data (unexposed) from BEIR IV (1988), Table 2A-10, p. 133: death rates of the male US population, surviving at least 30 years, applied to a birth cohort of 100,000. Exposure impact: advancement of certain fractions of deaths. For details of assumed mechanism see Table 2 (Additional file 2). Click here for file

Additional File 2 Two different exposure-response mechanisms compatible with the life table analysis in Table 1. Fractions of the unexposed deaths are advanced by 0 yr or 5 yr according to mechanism 1 and by 0 yr, 5 yr or 10 yr according to mechanism 2. The age distribution of all exposed deaths is identical under both mechanisms. The distribution of the true excess Years of Life Lost e-YLL differs between mechanisms and both diverge from e-YPLL (Table 1, see Additional file 1), whereas the totals agree. (advcm = advancement) Click here for file

Additional File 3 Life table analysis with calculation of excess Years of Potential Life Lost e-YPLL and true excess Years of Life Lost e-YLL for overall and lung cancer mortality (ICD9-162). Basic data (unexposed) from BEIR IV (1988), Table 2A-10, p. 133: overall and lung cancer death rates of the male US population, surviving at least 30 years applied to a birth cohort of 100,000. Exposure impact: advancement of factual and hypothetical lung cancer deaths by 5 years, mixture of advancements among deaths from all causes. It is assumed that the advancement of hypothetical lung cancer deaths leads to an excess of 50% of lung cancer deaths among exposed in each age category. The overall e-YLL for lung cancer are less than the number of exposed lung cancer deaths times 5 years. The e-YPLL for overall death and lung cancer death are determined according to Park et al. 2002. For all deaths e-YPLL must equal e-YLL, but e-YPLL is obviously biased for lung cancer death. Click here for file

Additional File 4 Excel sheet explaining the calculations in Additional files 1 and 2. Click here for file

Additional File 5 Excel sheet explaining the calculations in Additional file 3. Click here for file
