# Supplementary material for: Years of Life Lost due to exposure: Causal concepts and empirical shortcomings
Source: Epidemiol Perspect Innov. 2004 Dec 16;1:5. doi: 10.1186/1742-5573-1-5 (PMC545055; doi:10.1186/1742-5573-1-5)
Supplement: Additional File 1 — Life table analysis with calculation of excess Years of Potential Life Lost e-YPLL according to Park et al. 2002. Basic data (unexposed) from BEIR IV (1988), Table 2A-10, p. 133: death rates of the male US population, surviving at least 30 years, applied to a birth cohort of 100,000. Exposure impact: advancement of certain fractions of deaths. For details of assumed mechanism see Table 2 (Additional file 2). [file 1742-5573-1-5-S1.pdf]

Table 1: Life table analysis with calculation of excess Years of Potential Life Lost e-YPLL according to Park et al. 2002. Basic data (unexposed) from BEIR IV (1988), Table 2A-10, p. 133: death rates of the male US population, surviving at least 30 years, applied to a birth cohort of 100,000. Exposure impact: advancement of certain fractions of deaths. For details of assumed mechanism see Table 2.

Age in years	Population unexposed	No of Deaths unexposed	Life Expectation at Death in years, unexposed	Population exposed	No of Deaths exposed	Excess Deaths	e-YPLL
0-4	95218.73	0.00	70.35	95218.73	0.00	0.00	0.00
5-9	95218.73	0.00	65.35	95218.73	0.00	0.00	0.00
10-14	95218.73	0.00	60.35	95218.73	0.00	0.00	0.00
15-19	95218.73	0.00	55.35	95218.73	0.00	0.00	0.00
20-24	95218.73	0.00	50.35	95218.73	0.00	0.00	0.00
25-29	95218.73	0.00	45.35	95218.73	528.35	528.35	23961.01
30-34	95218.73	928.35	40.75	94690.38	948.62	25.42	1035.71
35-39	94290.38	1148.62	36.19	93741.76	1381.04	239.10	8652.82
40-44	93141.76	1681.04	31.76	92360.73	2532.44	865.50	27489.94
45-49	91460.73	2632.44	27.56	89828.29	3090.71	505.26	13922.40
50-54	88828.29	4090.71	23.64	86737.58	5468.21	1473.78	34846.00
55-59	84737.58	5968.21	20.06	81269.37	6814.23	1090.29	21867.40
60-64	78769.37	8314.23	16.83	74455.14	9996.22	2137.36	35978.90
65-69	70455.14	10996.22	14.02	64458.92	12331.36	2270.99	31843.30
70-74	59458.92	13331.36	11.63	52127.56	13391.34	1703.76	19813.20
75-79	46127.56	14391.34	9.64	38736.22	14146.45	2061.13	19859.47
80-84	31736.22	13646.45	8.13	24589.77	12521.41	1947.91	15840.18
85-89	18089.77	11021.41	8.02	12068.36	7306.48	-46.31	-371.19
90-94	7068.36	4306.48	7.72	4761.88	2882.71	-18.52	-142.95
95-99	2761.88	1682.71	6.95	1879.17	1157.50	12.59	87.55
100-104	1079.17	657.50	5.00	721.67	521.67	81.99	409.93
105+	421.67	421.67	0.00	200.00	200.00	0.00	0.00
Total		95218.73			95218.73		255093.66
