# Supplementary material for: Years of Life Lost due to exposure: Causal concepts and empirical shortcomings
Source: Epidemiol Perspect Innov. 2004 Dec 16;1:5. doi: 10.1186/1742-5573-1-5 (PMC545055; doi:10.1186/1742-5573-1-5)
Supplement: Additional File 2 — Two different exposure-response mechanisms compatible with the life table analysis in Table 1. Fractions of the unexposed deaths are advanced by 0 yr or 5 yr according to mechanism 1 and by 0 yr, 5 yr or 10 yr according to mechanism 2. The age distribution of all exposed deaths is identical under both mechanisms. The distribution of the true excess Years of Life Lost e-YLL differs between mechanisms and both diverge from e-YPLL (Table 1, see Additional file 1), whereas the totals agree. (advcm = advancement) [file 1742-5573-1-5-S2.pdf]

Table 2: Two different exposure-response mechanisms compatible with the life table analysis in Table 1. Fractions of the unexposed deaths are advanced by 0 yr or 5 yr according to mechanism 1 and by 0 yr, 5 yr or 10 yr according to mechanism 2. The age distribution of all exposed deaths is identical under both mechanisms. The distribution of the true excess Years of Life Lost e-YLL differs between mechanisms and both diverge from e-YPLL (Table 1), whereas the totals agree. (advcm = advancement)

Mechanism 1

Age in years	No of Deaths unexposed	No of Deaths exposed no advcm	No of Deaths exposed advcm = 5 yr	No of Deaths exposed total	e-YLL
25-29	0,00	0,00	528,35	528,35	2641,75
30-34	928,35	400	548,62	948,62	2743,08
35-39	1148,62	600	781,04	1381,04	3905,18
40-44	1681,04	900	1632,44	2532,44	8162,19
45-49	2632,44	1000	2090,71	3090,71	10453,55
50-54	4090,71	2000	3468,21	5468,21	17341,06
55-59	5968,21	2500	4314,23	6814,23	21571,14
60-64	8314,23	4000	5996,22	9996,22	29981,09
65-69	10996,22	5000	7331,36	12331,36	36656,79
70-74	13331,36	6000	7391,34	13391,34	36956,71
75-79	14391,34	7000	7146,45	14146,45	35732,25
80-84	13646,45	6500	6021,41	12521,41	30107,05
85-89	11021,41	5000	2306,48	7306,48	11532,42
90-94	4306,48	2000	882,71	2882,71	4413,53
95-99	1682,71	800	357,50	1157,50	1787,49
100-104	657,50	300	221,67	521,67	1108,36
105+	421,67	200	0,00	200,00	0,00
Total	95218,73	44200	51018,73	95218,73	255093,66
	no of deaths times advcm =		255093,66		

Mechanism 2

Age in years	No of Deaths unexposed	No of Deaths exposed no advcm	No of Deaths exposed advcm = 5 yr	No of Deaths exposed advcm = 10 yr	No of Deaths exposed total	e-YLL
25-29	0,00	0,00	508,35	20,00	528,35	2741,75
30-34	928,35	420	278,62	250,00	948,62	3893,08
35-39	1148,62	850	441,04	90,00	1381,04	3105,18
40-44	1681,04	990	742,44	800,00	2532,44	11712,19
45-49	2632,44	1800	1270,71	20,00	3090,71	6553,55
50-54	4090,71	2020	1648,21	1800,00	5468,21	26241,06
55-59	5968,21	4300	1714,23	800,00	6814,23	16571,14
60-64	8314,23	4800	5146,22	50,00	9996,22	26231,09
65-69	10996,22	5050	5281,36	2000,00	12331,36	46406,79
70-74	13331,36	8000	5091,34	300,00	13391,34	28456,71
75-79	14391,34	7300	6816,45	30,00	14146,45	34382,25
80-84	13646,45	6530	5891,41	100,00	12521,41	30457,05
85-89	11021,41	5100	1406,48	800,00	7306,48	15032,42
90-94	4306,48	2800	32,71	50,00	2882,71	663,53
95-99	1682,71	850	297,50	10,00	1157,50	1587,49
100-104	657,50	310	211,67	0,00	521,67	1058,36
105+	421,67	200	0,00	0,00	200,00	0,00
Total	95218,73	51320	36778,73	7120,00	95218,73	255093,66
	no of deaths times advcm =		183893,66	71200,00		
