# Supplementary material for: Years of Life Lost due to exposure: Causal concepts and empirical shortcomings
Source: Epidemiol Perspect Innov. 2004 Dec 16;1:5. doi: 10.1186/1742-5573-1-5 (PMC545055; doi:10.1186/1742-5573-1-5)
Supplement: Additional File 3 — Life table analysis with calculation of excess Years of Potential Life Lost e-YPLL and true excess Years of Life Lost e-YLL for overall and lung cancer mortality (ICD9-162). Basic data (unexposed) from BEIR IV (1988), Table 2A-10, p. 133: overall and lung cancer death rates of the male US population, surviving at least 30 years applied to a birth cohort of 100,000. Exposure impact: advancement of factual and hypothetical lung cancer deaths by 5 years, mixture of advancements among deaths from all causes. It is assumed that the advancement of hypothetical lung cancer deaths leads to an excess of 50% of lung cancer deaths among exposed in each age category. The overall e-YLL for lung cancer are less than the number of exposed lung cancer deaths times 5 years. The e-YPLL for overall death and lung cancer death are determined according to Park et al. 2002. For all deaths e-YPLL must equal e-YLL, but e-YPLL is obviously biased for lung cancer death. [file 1742-5573-1-5-S3.pdf]

Table 3: Life table analysis with calculation of excess Years of Potential Life Lost e-YPLL and true excess Years of Life Lost e-YLL for overall and lung cancer mortality (ICD9-162). Basic data (unexposed) from BEIR IV (1988), Table 2A-10, p. 133: overall and lung cancer death rates of the male US population, surviving at least 30 years applied to a birth cohort of 100,000. Exposure impact: advancement of factual and hypothetical lung cancer deaths by 5 years, mixture of advancements among deaths from all causes. It is assumed that the advancement of hypothetical lung cancer deaths leads to an excess of 50% of lung cancer deaths among exposed in each age category. The overall e-YLL for lung cancer are less than the number of exposed lung cancer deaths times 5 years. The e-YPLL for overall death and lung cancer death are determined according to Park et al. 2002. For all deaths e-YPLL must equal e-YLL, but e-YPLL is obviously biased for lung cancer death.

Age in years	Population unexposed	No of Deaths unexposed	No of Deaths ICD9-162 unexposed	Life Expectation at Death in years, unexposed	Population exposed	No of Deaths exposed	No of Deaths ICD9-162 exposed	Excess Deaths overall	e-YPLL overall	Excess Deaths ICD9-162	e-YPLL ICD9-162
0-4	95218.73	0.00	0.00	70.35	95218.73	0.00	0.00	0.00	0.00	0.00	0.00
5-9	95218.73	0.00	0.00	65.35	95218.73	0.00	0.00	0.00	0.00	0.00	0.00
10-14	95218.73	0.00	0.00	60.35	95218.73	0.00	0.00	0.00	0.00	0.00	0.00
15-19	95218.73	0.00	0.00	55.35	95218.73	0.00	0.00	0.00	0.00	0.00	0.00
20-24	95218.73	0.00	0.00	50.35	95218.73	0.00	0.00	0.00	0.00	0.00	0.00
25-29	95218.73	0.00	0.00	45.35	95218.73	320.53	8.81	320.53	14536.23	8.81	399.63
30-34	95218.73	928.35	5.87	40.75	94898.20	1350.00	40.70	424.77	17308.67	34.84	1419.69
35-39	94290.38	1148.62	27.13	36.19	93548.20	1736.00	133.26	596.42	21583.84	106.34	3848.29
40-44	93141.76	1681.04	88.84	31.76	91812.20	2189.00	335.04	531.96	16896.10	247.47	7860.26
45-49	91460.73	2632.44	223.36	27.56	89623.20	3379.00	659.41	799.45	22028.89	440.53	12138.88
50-54	88828.29	4090.71	439.60	23.64	86244.20	4670.00	1046.55	698.29	16510.35	619.74	14652.98
55-59	84737.58	5968.21	697.70	20.06	81574.20	7259.00	1475.07	1513.59	30357.36	803.41	16113.64
60-64	78769.37	8314.23	983.38	16.83	74315.20	11215.00	1771.61	3370.92	56743.68	843.84	14204.64
65-69	70455.14	10996.22	1181.07	14.02	63100.20	12710.00	1802.55	2861.70	40125.99	744.77	10442.97
70-74	59458.92	13331.36	1201.70	11.63	50390.20	14470.00	1434.54	3171.95	36887.01	416.12	4839.13
75-79	46127.56	14391.34	956.36	9.64	35920.20	13860.00	862.89	2653.25	25564.68	118.16	1138.47
80-84	31736.22	13646.45	575.26	8.13	22060.20	10250.00	323.71	764.20	6214.40	-76.16	-619.29
85-89	18089.77	11021.41	215.81	8.02	11810.20	8134.00	126.49	938.49	7522.35	-14.41	-115.48
90-94	7068.36	4306.48	84.32	7.72	3676.20	2150.00	49.42	-89.77	-692.76	5.57	42.96
95-99	2761.88	1682.71	32.95	6.95	1526.20	880.00	19.31	-49.86	-346.69	1.10	7.68
100-104	1079.17	657.50	12.87	5.00	1070.00	412.20	12.39	-239.71	-1198.55	-0.38	-1.90
105+	421.67	421.67	8.26	0.00	657.80	234.00	0.00	-423.80	0.00	-12.88	0.00
Total		95218.73	6734.49			95218.73	10101.74	17842.39	310041.56	4286.88	86372.55
										e-YLL<	50508.68
										bias >	35863.87
										relative bias / % >	71.01
